# Quality Performance Evaluation of the Largest COVID-19-Designated Intensive Care Unit in the Western Region of Saudi Arabia

**DOI:** 10.7759/cureus.39800

**Published:** 2023-05-31

**Authors:** Ayman Morish, Abdulrahman Alsaigh, Ehab Almaghrabi, Huda Alenzi, Nizar M Ahmed, Ali AlAhdal

**Affiliations:** 1 Critical Care Medicine, Mediclinic Al Murjan Hospital, Jeddah, SAU; 2 Integrated Care Outcome Management, Makkah Health Affairs, Makkah, SAU; 3 Forensic Medicine Department, Makkah Health Affairs, Makkah, SAU; 4 Nursing Administration, King Saud Medical City, Riyadh, SAU; 5 Integrated Healthcare Outcomes Management, Makkah Health Affairs, Makkah, SAU

**Keywords:** covid-19 outcome predictor, risk of covid-19 mortality, impact of covid-19, covid-19 infection, covid-19 pandemic, covid-19 in saudi arabia, icu patients, medical icu, covid-19 outbreak, covid 19

## Abstract

Introduction

Mortality is generally higher among patients with coronavirus disease 2019 (COVID-19) than non-COVID-19 patients, especially critically ill patients. The Acute Physiology and Chronic Health Evaluation IV (APACHE IV) predicts mortality rate (MR); however, it was not designed for COVID-19 patients. Multiple indicators have been utilized in healthcare to measure the performance of intensive care unit (ICU) departments, including length of stay (LOS) and MR. The 4C mortality score was recently developed using the ISARIC WHO clinical characterization protocol. This study aims to evaluate intensive care unit performance using LOS, MR, and 4C mortality scores at East Arafat Hospital (EAH), Makkah region, which is considered the largest COVID-19-designated intensive care unit in the Western region of Saudi Arabia.

Materials and methods

A retrospective observational cohort study was conducted on data from patients’ records during the COVID-19 pandemic, from March 1, 2020, to October 31, 2021, at EAH, Makkah Health Affairs. Data to calculate LOS, MR, and 4C mortality scores were collected from the eligible patients' files by a trained team. Demographic (age and gender) and clinical data on admission were collected for statistical purposes.

Results

A total of 1298 patient records were included in the study; 417 (32%) of the patients were female and 872 (68%) were male. The cohort included 399 deaths (total MR=30.7%). Most deaths occurred in the 50-69-year age group, with significantly more deaths among female patients than male patients (p=0.004). A significant association was found between the 4C mortality score and death (p<0.000). Furthermore, the mortality odds ratio (OR) was significant (OR=1.3, 95% CI=1.178-1.447) for each added 4C score.

Conclusion

Our study metrics regarding LOS were generally higher than most internationally reported values and slightly lower than locally reported values. Our reported MR was comparable with overall published MRs. The ISARIC 4C mortality score was highly compatible with our reported MR between scores 4 and 14; however, the MR was higher for scores 0-3 and lower for scores ≤15. The overall performance of the ICU department was considered generally good. Our findings are helpful for benchmarking and motivating better outcomes.

## Introduction

The intensive care unit (ICU) is an integral part of any acute care healthcare facility as it provides highly specialized and close monitoring for critically ill patients with life-threatening conditions [1]. Mortality is generally higher in patients with coronavirus disease 2019 (COVID-19) than in non-COVID-19 patients, especially critically ill patients. This disease results from infection with the severe acute respiratory syndrome coronavirus 2 (SARS-CoV-2) and has a high mortality rate (MR), with death predominantly caused by respiratory failure. Multiple indicators have been utilized to measure the performance of ICU departments, including length of stay (LOS) and MR, both of which are measures of department effectiveness [2]. The enhanced model to predict hospital mortality among ICU patients (Acute Physiology and Chronic Health Evaluation [APACHE] IV) can predict MR but was not designed for COVID-19 patients [3]. Recently, the 4C Mortality Score was developed using the International Severe Acute Respiratory and Emerging Infection Consortium-World Health Organization (ISARIC WHO) Clinical Characterization Protocol. The model is a risk stratification tool that predicts mortality for hospitalized COVID-19 patients. These scores were developed to be used only by physicians to aid in their clinical decision-making; however, physicians should also rely on their knowledge and experience [4].

Commonly available parameters comprise the 4C predictive score [5]. The parametric predictive values should be measured at the time of hospital presentation for suspected community-acquired cases or at the first evaluation for COVID-19 for suspected nosocomial cases. During the COVID-19 pandemic, East Arafat Hospital (EAH) was designated to serve the citizens of Makkah City in the Western region of Saudi Arabia as a COVID-19 center. This hospital is one of the seasonal hospitals that operate only during the mass gathering of Hajj. EAH was prepared and launched to serve exclusively from March 2020 to October 2021 to address the urgent need to increase the region's ICU bed capacity due to the COVID-19 pandemic.

This study evaluates the performance of the ICU at EAH (from March 2020 to October 2021), Makkah region, which is considered the largest COVID-19-designated ICU in the Western region of Saudi Arabia, with a capacity of 50 ICU beds.

## Materials and methods

A retrospective observational cohort study was conducted on patients’ records during the COVID-19 pandemic at EAH, Makkah Health Affairs, from March 1, 2020, to October 31, 2021. The institutional review board of Makkah Health Affairs (no. H-02-K-076-0822791) approved this study. The study included all records within the inclusion criteria of the hospital, which was designated for patients with COVID-19 infection confirmed by laboratory analysis of a nasopharyngeal swab.

Inclusion criteria

The study included all laboratory-confirmed COVID-19 patients, through the polymerase chain reaction (PCR) technique, who were admitted to the ICU with a known hospital outcome. They include male, non-pregnant female, age ≥18 years old, free medical history from human immunodeficiency virus or pulmonary tuberculosis infection, unknown history of malignancy, and inactive do-not-resuscitate orders.

Exclusion criteria

Patients aged <18 years, pregnant females, and patients with known human immunodeficiency virus or pulmonary tuberculosis infection, a history of malignancy, or active do-not-resuscitate orders were excluded.

Data collection

Data to calculate LOS, MR, and 4C mortality scores were collected from the eligible patients' files by a trained team from the hospital’s health information system. Demographic (age and gender) and clinical data on admission were collected for statistical purposes. The clinical data included date of admission, Glasgow Coma Scale, respiratory rate (RR), oxygen saturation, number and type of comorbidities (hypertension, body mass index [BMI], diabetes mellitus, congestive heart failure, renal diseases, asthma, chronic obstructive pulmonary disease, and active malignancies), sodium, potassium, urea, C-reactive protein (CRP), lymphocyte count, mechanism of oxygen supply on admission (high-flow nasal cannula [HFNC], face mask [FM], bilevel positive airway pressure [BiPAP], continuous positive airway pressure [CPAP]), fraction of inspired oxygen (FiO_2_), and patient outcome (deceased or alive).

Statistical method

The 4C score was calculated, and its performance was evaluated against the known outcomes of the patients. All the represented data were non-parametric (using the Anderson-Darling test with a p-value >0.05) and are presented as median and interquartile (Q1-Q3) ranges. We used binary logistic regression analysis for the outcome to predict the probability model of death against continuous and categorical independent variables. Statistical analysis was performed based on a 95% confidence level and 5% marginal error with a significance level of 0.05 (p≤0.05) using Minitab® 19.2020 software (Minitab Ltd., Coventry, UK).

## Results

A total of 1298 patients' records were included in the study, including a total of 399 deaths (total MR=30.7%). A binary logistic regression analysis of the data was used to understand their effect on the outcomes (Table 1). The Hosmer-Lemeshow test indicated that the model fit the data well (chi-square=11.3, p=0.741).

**Table 1:** Patient characteristics on admission with treatment outcomes. ^#^Using binary logistic regression. WBC: white blood cells, HCT: hematocrit test CRP: C-reactive protein, Na: sodium, K: potassium.

Characteristic	Overall	Died	Survived	p-value^#^
Age				
<50	333	62	271	0.324
50–69	746	219	527	
70–79	161	69	92	
≥80	100	48	52	
Gender				
Female	417	157	260	0.004
Male	872	242	630	
White blood cells				
4.5–11/µL	138	44	94	0.274
11↑/µL	1200	355	845	
HCT				
41–53%	501	110	391	0.005
↓41%	830	282	548	
53%↑	7	7	0	
Na				
136–145	747	209	538	<0.000
136↓	265	61	204	
↑145	303	129	174	
K				
3.7–4.5	822	228	594	0.74
3.7↓	277	85	192	
↑4.5	237	86	151	
CRP mg/L				
69–97	1017	298	719	0.054
69↓	104	42	62	
↑97	141	58	83	
Urea mmol/L				
6.3–11.4	726	195	531	0.477
6.3↓	260	65	195	
↑11.4	244	141	103	
Creatinine				
79–138	712	190	522	0.032
79↓	293	75	218	
↑138	339	141	198	

The median age of the patients was 57 (49-66) years, and the odds of death decreased with younger age by 0.9904 (95% confidence interval [CI] = 0.9761-1.0096). Most deaths occurred in the 50-69-year-old age group. Four hundred seventeen (32%) of the patients were female, and 872 (68%) were male, with significantly more deaths among female patients than male patients (p=0.004). The odds ratio (OR) of death outcome was significant among male patients compared with female patients (OR = 0.55; 95% CI=0.39-0.78). A significant association was found between death and increased sodium (p<0.000, OR=1.0364, 95% CI=1.016-1.057), CRP (p=0.029, OR=0.996, 95% CI=0.9926-0.9996), creatinine (p=0.011, OR=1.0029, 95% CI=1.007-1.05), FiO_2_ (p=0.009, OR=5.6102, 95% CI=1.5449-20.3744), and LOS (mean=15, p<0.000, OR=1.0497, 95% CI=1.0351-1.0644). In contrast, a significant inverse association was detected between death and hematocrit (p=0.005, OR=0.969, 95% CI=0.94-0.99) and RR (p<0.000, OR=0.9583, 95% CI=0.9361-0.9811). Moreover, the death outcome was significantly less common among patients on FM (p=0.003, coefficient = −1.453) and HFNC (p=0.034, coefficient = −1.101) as initial modes of oxygen supply on admission.

**Figure 1:**
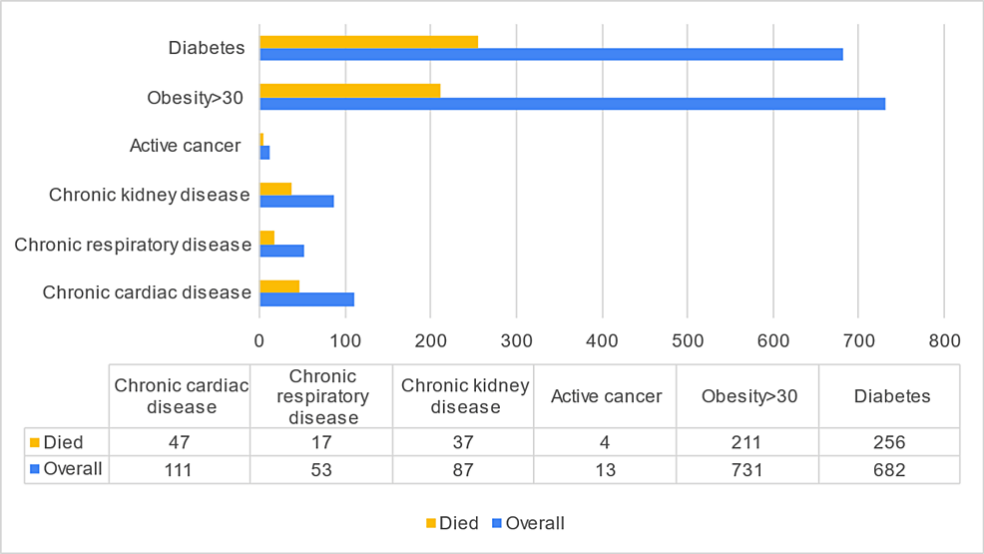
Distribution of death cases according to the type of comorbidity.

There was no statistically significant association between the outcomes and the types of comorbidities or BMI. A summary of the death case distribution according to the type of comorbidity is presented in Figure 1. Similarly, no association was found between death and oxygen saturation, potassium, urea, or other mechanisms of oxygen supply on admission (i.e., BiPAP, CPAP).

**Figure 2:**
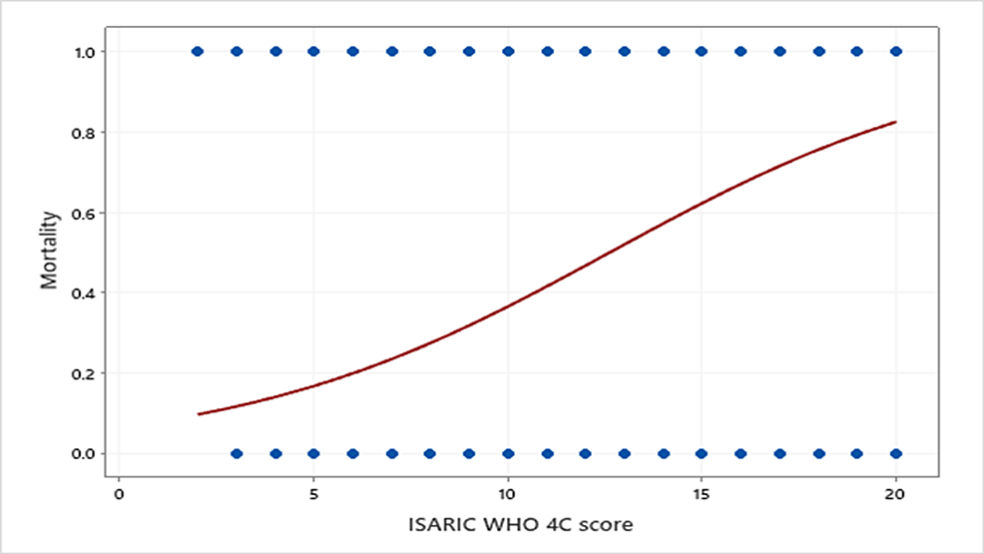
Binary logistic regression showing the relationship between ISIARIC WHO 4C score and mortality. ISARIC: International Severe Acute Respiratory and Emerging Infection Consortium, WHO: World Health Organization.

Using binary fitted plot line logistic regression analysis, we evaluated the relationship between the 4C score as a predictor and mortality (deceased or alive) (Figure 2); a significant association was found between these variables (p<0.000), and the OR of mortality was significant for each added 4C score (OR=1.3, 95% CI=1.178-1.447). The MR in the ICU according to the 4C score range is presented in Table 2.

**Table 2:** Actual mortality rate in the intensive care unit according to 4C score.

4C score range	4C risk group	Frequency	Deceased	Mortality rate (%)
0–3	Low	16	3	18.75
4–8	Intermediate	436	84	19.26
9–14	High	538	241	44.78
15≤	Very high	108	71	65.74

## Discussion

Our retrospective study aimed to evaluate the performance of the largest COVID-19-designated ICU in the Western region of Saudi Arabia from March 2020 to October 2021. The findings revealed that the total MR was 30.7%, males comprised the majority of COVID-19 patients admitted to ICU, the median LOS was 13 (7-20) days, and most of the patients' 4C scores ranged from intermediate (436) to high (538).

The total MR reported in our study was comparable to locally and globally reported MRs. A report from King Saud Medical City (KSMC), Riyadh, Saudi Arabia, stated an MR of 31.1% among 352 COVID-19 ICU patients [6]. Chang et al. conducted a meta-analysis of studies from more than five countries, and they reported a total MR of 28.3% for ICU-admitted COVID-19 patients [7]. However, a local study in Riyadh by Aleterby et al. reported a slightly higher MR of 38% [8], and early COVID-19 reports from Wuhan (China) and Seattle (USA) found much higher rates (78% and 85%, respectively) [9,10].

In line with our findings, Chang et al. reported a positive association between death and patient age, CRP, creatinine, FiO_2_, and LOS [7]. Though the KSMC study reported no significant association between death and creatinine level, the study corroborated our finding of a lack of association between death and BMI [6]. Moreover, Chang et al. reported increased mortality associated with male gender (pOR=1.8, 95% CI=1.25-2.59), while we observed a significant positive association between death and female gender (p=0.004); the OR to die following ICU admission for COVID-19 and being male was 0.55 (95% CI=0.39-0.78). We did not define any obvious correlated factors for the difference between genders, considering the confounding variables.

We observed that more male patients (n=872) were admitted to the ICU than female patients (n=417). As Zhang et al. indicated in their study on the risk and protective factors of COVID-19 morbidity, severity, and mortality, male patients were prone to COVID-19 infection (risk ratio=1.08, 95% CI=1.03-1.12) [11]. Furthermore, in the Lombardy region of Italy, men (79.9% out of 3988 patients) were admitted more frequently than women [12], while at the KSMC in Riyadh, Saudi Arabia, 87.2% of 352 critically ill COVID-19 patients were males [6]. Several influencing factors were suggested, including hormonal differences between males and females and their effects on the inflammatory response, receptor levels, and lifestyles [13].

There was a discrepancy between the mean LOS in the ICU for COVID-19 patients that we observed in our study and the LOS that other studies reported globally. In a study conducted in Argentina [14], we reported a median LOS of 13 (7-20) days, while the mean LOS was 6.75 days. At Adiyaman University Education and Research Hospital in Turkey, the median LOS was lower, at four (0-51) days [15]. In contrast, Alharthy et al. published a median LOS of 18 (9-29) days in KSMC, which was higher than our reported LOS [6]; however, a different research group reported a mean ± standard deviation LOS of 8.1 ± 7.2 days for KSMC [8]. These contrasting reports from the same facility are primarily attributed to the different times at which the studies were conducted.

The ISARIC 4C mortality score was initially developed for the UK population [4]. A study was conducted in KSMC, Riyadh, Saudi Arabia, to validate the ISARIC 4C mortality score in different populations, and it concluded the same outcomes as the original research in the UK [8]. Our report revealed a higher actual MR for the low-risk category (18.75) than both reports, while the MRs for the intermediate- and high-risk groups were comparable. However, the very high-risk group in our study had a lower MR compared with the two studies mentioned above.

Limitations

This was a single-center study with a limited population; therefore, the findings cannot be generalized to all COVID-19 patients in the region or the country.

## Conclusions

The LOS reports from our cohort were higher than most internationally reported LOSs and slightly lower than locally reported LOSs. However, our reported actual MR was comparable to the overall published MRs. The ISARIC 4C mortality score was highly compatible with our reported actual MR between scores 4 and 14, higher for scores 0-3, and lower for scores ≥ 15. Therefore, the overall performance of the ICU department evaluated in this study was generally good. Our findings are helpful for benchmarking and motivating better outcomes.
